# Botulinum toxin therapy in Parkinson disease-related lower limb dystonia. An 8 year retrospective review

**DOI:** 10.1016/j.prdoa.2024.100260

**Published:** 2024-06-10

**Authors:** Antonia Schonwald, Katherine Amodeo, Victoria Levy, Fabio Danisi

**Affiliations:** a New York Medical College, Valhalla, NY, USA; b Westchester Medical Center Health Network, Neurology, Poughkeepsie, NY, USA; c NewYork-Presbyterian Healthcare System Inc, Anesthesiology, New York, NY, USA

**Keywords:** Parkinson disease, Limb dystonia, Botulinum toxin, Foot dystonia, Dystonia progression, Therapeutics

## Abstract

**Background:**

Lower extremity dystonia (LED) is a frequent complication of Parkinson disease (PD). Treatment with botulinum neurotoxinA (BoNTA) over 8 years was retrospectively reviewed.

Cases

14 patients with LED received an average of 3.86 injections (1–8). Mean interval was 40 weeks (median of 25). Average dose was 182 units. Injections were well-tolerated. Using a 6 point scale, there was an average of 3.37 point improvement in disability after each session, with average duration of 28.56 weeks (median 11 weeks). After mean follow-up of 101 weeks, disabling dystonia was not present in 11 of 14 patients.

**Conclusions:**

Botulinum toxin is safe and effective in PD related LED. Good response to the first two injection sessions was significantly associated with greater likelihood of long-term response. Assertive BoNTA dosing may lead to sustained remission of symptoms. As natural history of LED in PD has not been reported, prospective placebo-controlled studies are needed.

## 1 Introduction

Dystonia is a complex, highly variable neurological movement disorder characterized by involuntary muscle contractions leading to abnormal postures. Lower extremity dystonia (LED) is a common finding in patients with Parkinson’s Disease (PD) [1], [2]. The involvement of the lower limb is more common than the upper limb [3]. While dystonia of all types is estimated to be present in about 39 % of patients with PD, particularly in younger age groups, [4] the incidence and prevalence of LED is unknown [5]. PD patients can develop a fluctuating response to medications, defined as ON when medications are effective, and OFF when PD symptoms re-emerge as medications wane. PD-related dystonia can be an early or later manifestation of PD itself (OFF dystonia), or can be secondary to PD medications (ON dystonia) [3], [4], [5].

Dystonia can be disabling due to interference from abnormal posture and pain associated with muscle contractions and malposition of the affected body part. LED typically occurs on the side more affected by PD and can present in a task-specific manner, particularly by attempts to walk. LED can interfere with activities such as walking, and thus hinder patients’ ability to exercise.

OFF dystonia is often successfully treated with PD medications, including levodopa, dopamine agonists, amantadine, anticholinergics; or use of benzodiazepines or other muscle relaxants. Similarly, ON dystonia can be successfully treated by reduction of PD medications, use of muscle relaxants, amantadine, or dose fragmentation [6]. Deep brain stimulation (DBS) is known to alleviate both ON and OFF dystonic symptoms in PD [7].

In many patients, however, control of dystonic symptoms cannot be achieved with medication adjustment alone. Botulinum toxin (BoNT) injections have been shown to be a safe and effective therapy for focal dystonia in PD [8], [9], [10], [11], [12], [13].

In addition to the direct effect of BoNT on selective chemodenervation of the overactive muscle, there is indirect action of BoNT treatment on the dystonic sensorimotor network through modulation of motor sequence planning and correction of the maladaptive plastic changes within the sensorimotor cortex [14].

It has been the authors' observation that lower extremity dystonic symptoms tend to resolve after a few injection sessions, unlike other PD symptoms, which are inexorably progressive. This has prompted us to perform an analysis of our experience treating PD patients with disabling LED to highlight patient characteristics, response to therapy, and identify any features that might predict the eventual resolution of symptoms.

In this paper, we will review our experience over 8 years in treatment of disabling LED. Clinical characteristics, dosing, and natural history will be reviewed.

## 2 Case series

After IRB approval (NYMC Protocol #16859), medical records were reviewed of PD patients followed by one of the authors (FD). The patients who received BoNT injections to the lower extremities from October 2015 through June 2023 were identified by two independent reviewers (AS and VL). The reviewers identified the charts through diagnosis codes for PD and treatment codes for BoNT. The charts were then individually inspected for the type of dystonia as dystonia was often not coded but its presentation and treatment were described within the charts. Dystonia that did not affect the lower extremities was not included for data analysis. Patients who received at least one BoNT injection were included in the study and followed until the end of the study period or until death. The injections included both onabotulinumtoxinA (Botox® Allergan) and incobotulinumtoxinA (Xeomin® Merz), as they are considered equivalent both for efficacy and unit dosing. All injections were guided through electromyography, although in some, electrical stimulation and ultrasound were also used to identify affected muscles. While the goal of this retrospective review was to include all PD patients with disabling LED who were treated with BoNT regardless of the outcome, we deemed it necessary to exclude injection sessions where more than 50 % of the dose was given to the thigh muscles. This was done to exclude cases of dystonic hamstring shortening, which tend to be present in older, more advanced PD patients [15], [16]. We reviewed the dose and response to each injection session and number of injection sessions for each individual. We also ascertained the duration of follow-up after the last recorded injection session. Lastly, we verified if disabling LED had returned after the last injection session.

Given the retrospective nature of this review, information on therapies that preceded BoNT injections was not extensively evaluated due to limited data availability within the patient charts. However, all patients with LED were treated with BoNT only if (1) they failed more conservative therapies or (2) had severe functional disabilities due to pain and/or interference with station and gait. Furthermore, given the limitations in the coding classifications within the electrical medical records system, the prevalence and incidence of LED was not identified within our patient population. Mild and moderate LED is often responsive to oral antiparkinsonian therapy and does not necessitate BoTN injections. This review focuses on assessing the responsiveness of disabling LED to BoTN. However, the percentage of patients who met the PD diagnosis and received BoTN for LED is described in the results.

## 3 Results

Patient characteristics and clinical timelines are presented in Table 1. From a total of 320 patients who met the PD diagnosis, 14 patients (4.4 %) were identified to have received BoTN for LED. 54 injection sessions were identified across 14 individual patients aged 43–92 at the time of their initial injection session. The onset of disabling LED ranged from 48-81 years of age (median 61). In all of these patients, modification of dosing or timing of oral medications for PD failed to improve disabling LED. Eight patients received Botox, 5 received Xeomin, and one patient received 7 Botox injections, then Xeomin for the eighth and last injection. We assumed a 1:1 dose conversion between Botox and Xeomin [17], [18]. Each patient underwent an average of 3.86 injections (1–8). The mean interval between injections was 40.1 weeks, with a median of 25.1 weeks. The average dose of BoNTA (Botox or Xeomin) per injection session was 182 units. All injections were done under electromyographic guidance. Ultrasound guidance was used in two patients. The most commonly selected muscles were the Flexor Digitorum Longus (11 patients), Tibialis Posterior (9 patients), and Flexor Digitorum Brevis (8 patients). Table 1 summarizes the clinical characteristics, number of injection sessions, muscles injected, average dose, and analysis of the most frequently injected muscles across the 14 patients in our cohort.

**Table 1:** Patient characteristics and clinical timelines. The table includes the age of the patient at PD onset, time from PD onset to disabling dystonia (months), time from onset of disabling dystonia to initial BoNT treatment (months), total number of BoNT treatments, and average BoNT treatment interval (weeks). Injection details are summarized per individual patient and across all treatments in the cohort.

patient	Age at PD onset (years)	PD onset to intractable dystonia (months)	Disabling dystonia to BoNT treatment (months)	Number of injection sessions	Muscles injected	Average dose across injections (u)	Average BoNT treatment interval (weeks)	Recurrence of dystonia at last followup		*number of times each muscle was selected for injection in the* *57 injection sessions across 14 patients*	
1	32	128	1	2	EHL EDL EDB	55	23	No		*FDL = flexor digitorum longus*	*FDL = 11*
2	58	46	5	4	FDL TP SOL EHL EDL EDB EHB	74	64	No		*TP = tibialis posterior*	*TP = 9*
3	67	42	5	8	FDL TP FDB SOL FHL EDB FHB	145	23	No		*FDB = flexor digitorum brevis*	*FDB = 8*
4	55	36	4	5	FDL TP FDB FHL FHB	104	40	No		*SOL = soleus*	*SOL = 6*
5	85	78	5	3	EHL EDL EDB	110	16	No		*EHL = extensor hallucis longus*	*EHL = 6*
6	74	52	2	4	FDL FDB	59	25	No		*EDL = extensor digitorum longus*	*EDL = 5*
7	63	110	3	1	TP EHL TA	150	N/A	No		*FHL = flexor hallucis longus*	*FHL = 5*
8	50	168	15	5	FDL TP SOL MG TA	248	24	Yes		*EDB = extensor digitorum brevis*	*EDB = 4*
9	72	88	4	1	FDL FDB SOL EDL	60	N/A	Yes		*FHB = flexor hallucis brevis*	*FHB = 3*
10	46	118	7	4	FDL TP FDB SOL FHL FHB LG	148	109	No		*LG = lateral gastrocnemius*	*LG = 2*
11	58	78	1	2	FDL TP FDB EHL ADH	165	14	No		*MG = medial gastrocnemius*	*MG = 2*
12	68	129	5	8	FDL TP SOL FHL MG LG	260	24	Yes		*TA = tibialis anterior*	*TA = 2*
13	45	36	26	7	FDL TP FDB FHL	180	15	No		*ADH = adductor hallucis*	*ADH = 1*
14	50	20	27	3	FDL FDB EHL EDL	93	49	No		*EHB = extensor hallucis brevis*	*EHB = 1*

Other than injection site pain (typically mild-moderate), the only significant side effect was ankle instability due to weakness, prompting that patient to stop receiving BoNTA injections (1/14, 7.1 %). Two patients stopped due to loss of efficacy: one after 8 injection sessions and one patient after one injection session (2/14, 14.2 %).

A six-point semi-quantitative outcome scale was used before each injection session (0: no disability or functional impairment; 1: mild; 2: mild to moderate; 3: moderate; 4: moderate to severe; 5: severe disability or functional impairment). Scoring was based on patient reporting and the examiner’s assessment. This analysis is performed routinely in the clinic before all BoNTA injections.

The time interval from symptom onset to the first botox injections was on average 7.64 months.

There was an average of 3.37 point improvement in disability/functional impairment after each injection session. The average BoNTA injection benefit duration is 28.56 weeks, with a median of 11 weeks.

The mean follow-up from the last injection to the last observation point is 101 weeks (range 9–520 weeks, median 35 weeks). The last observation point was determined by chart review based on the most recent office note. Disabling dystonia was not present in 11 of the 14 patients at the last follow-up.

In clinical practice, it typically takes more than one injection session to achieve optimal results. Hence, to measure the treatment responsiveness, we examined changes in disability following the first two injection sessions. We utilized the six-point semi-quantitative outcome scale described above. To assess the response to each injection, we subtracted the disability score given after the treatment from the one given before the treatment (scale 0–5). A score of 5 indicates the highest possible improvement in outcome following treatment, while 0 indicates no improvement. No patient experienced a negative outcome, such as a further impairment in disability following treatment. The average response for the first injection was 3.86, while for the second was 3.66.

In our cohort, good response to the first two injection sessions was significantly associated with a greater likelihood of long-term response via unpaired *t*-test. The long-term response was defined as patients who had no return of disabling dystonia after the last injection. On the other hand, the time interval between the onset of disabling symptoms and treatment with botulinum toxin was not associated with a significantly higher likelihood of long-term response.

Following BoNT injection there was no signal that there were significant changes in medication regimen. Of note, bilateral subthalamic DBS was performed in one patient who had full remission of LED after first BoNT injections performed 10 months prior. After DBS, there was no recurrence of LED. (Patient 6, Table 1).

## 4 Discussion

Botulinum toxin injections were overall very effective and well tolerated. The magnitude of clinical improvement is 3.37 points using a six-point scale. Improvement of symptoms was even greater after the first two injection sessions (3.86 and 3.66 points, respectively). The most common side effects noted were transient injection site pain. Only one patient stopped due to side effects.

Dystonic symptoms did not return after relatively few injection sessions in 11 of the 14 patients in our cohort.

To our knowledge, the natural history of PD-related LED has not been described, and this phenomenon has not been discussed. This study suggests that early, effective treatment of LED increases the chances of full remission in a condition where every other symptom is slowly but inexorably progressive.

Several lines of evidence point to better response when treatment is initiated soon after dystonia symptom onset across various etiologies and various treatment modalities [19], [20], [21], [22], [23], [24]. Our results may add to the body of evidence that dystonia is a disorder of neural networks and neuronal plasticity with exaggerated LTP. This underscores the usefulness of early intervention in dystonia, regardless of etiology. It also suggests that repeat injection sessions are not likely to be beneficial if there is not a robust response to the first two sessions.

We theorize that a more aggressive dosing regimen might lead to better short- and long-term outcomes, including full remission of dystonic symptoms. This can only be determined via a double-blind study with a long observation period. Regardless, more aggressive dosing in the first injection sessions is more easily tolerated in muscles such as the toe flexors and foot inverters. Thus, higher initial doses can be attempted in the distal lower extremity than in other parts of the body where excessive toxin-induced weakness of injected muscles or neighboring muscles could be disabling.

These findings further suggest that while some patients with LED may require a brief course of BoNT therapy and achieve complete resolution, others may require alternative or advanced therapies. Future work may include looking more closely at distinguishing factors (ie, DATSCAN results, genetic profiles) in those with dystonia responsive to a brief course of BoNT versus those who are more refractory so that we can better identify responders. Prospective placebo-controlled studies across multiple injection sessions will be helpful to better characterize the natural history of LED and response to BoNT therapy.

## 5 Disclosures

No specific funding was received for this work. The authors declare that there are no conflicts of interest relevant to this work. The authors declare that there are no additional disclosures to report.

## 6 Ethical compliance statement

All data pertaining to this report have been collected following IRB approval: New York Medical College Protocol #16859 (Retrospective chart review of patient outcomes after treatment with botulinum toxin for Parkinson-related lower limb dystonia).

Chart review data was anonymized to protect PHI. Being a retrospective review, informed patient consent was not necessary for this work. We confirm that we have read the Journal’s position on issues involved in ethical publication and affirm that this work is consistent with those guidelines.

## CRediT authorship contribution statement

**Antonia Schonwald:** Writing – review & editing, Validation, Software, Project administration, Methodology, Investigation, Formal analysis, Data curation. **Katherine Amodeo:** Writing – review & editing, Validation, Methodology, Conceptualization. **Victoria Levy:** Writing – review & editing, Software, Methodology, Formal analysis, Data curation. **Fabio Danisi:** Research project Conception, Organization, Execution. Statistical Analysis Design, Review and Critique. Manuscript Preparation: Writing of the first draft, Review and Critique.

## Declaration of competing interest

The authors declare that they have no known competing financial interests or personal relationships that could have appeared to influence the work reported in this paper.
